# Deformation, Failure, and Acoustic Emission Characteristics under Different Lithological Confining Pressures

**DOI:** 10.3390/ma15124257

**Published:** 2022-06-15

**Authors:** Shuo Wu, Guangpeng Qin, Jing Cao

**Affiliations:** 1 College of Resource, Shandong University of Science and Technology, Tai’an 271000, China; skdyjsws@163.com; 2 National Engineering Laboratory for Coalmine Backfilling Mining, Shandong University of Science and Technology, Tai’an 271000, China; 3 College of Finance and Economics, Shandong University of Science and Technology, Tai’an 271000, China

**Keywords:** rock mechanics, compression test, acoustic emission, ambient pressure effect, rupture precursor

## Abstract

When a temporary support is used to control new surrounding rock in a deep mining roadway, the new surrounding rock is supported by the working resistance of the temporary support. In this study, the influence of deep well boring roadway deformation and rock failure characteristics under different surrounding pressure was investigated. In this paper, for each confining pressure, we experimentally identified the stress-strain, strength, and acoustic emission characteristics of the rocks. The results show that: the surrounding pressure has a significant effect on the damage deformation characteristics of the rock, and the change of the surrounding pressure directly affects the strength, damage form and elastic modulus of the rock; the strength limit of the rock increases with the surrounding pressure, and the damage form of the rock gradually changes to ductile damage with increase of the surrounding pressure; the elastic modulus of the rock increases non-linearly with the increase of the surrounding pressure. The acoustic emission signal of a rock can be divided into three stages: calm, sudden increase, and destruction. The acoustic emission ringing count rate increases suddenly and reaches a peak before the main fracture. Therefore, a sudden increase in the acoustic emission value can be considered a precursor to rock destruction.

## 1. Introduction

With the gradual depletion of shallow resources, there is an increasing need for deep energy mining. As the most important pillar industry in China, coal mining continues to deepen [1]. Rock underground engineering is generally buried at great depths, and the crossing formations, ground stress, and transmission of the underground structure are complex. Under a state of high ground stress, deep interaction relationships are enhanced. Rock disturbances caused by excavation of the upper rock body become more complex, and disasters are more frequent. Studies have shown that the mechanical response mechanisms of rock change with depth [2]. Deng et al. [3,4] conducted an indoor triaxial compression test on marble from the Jinping Grade II Hydropower Station in southwest China, and used maximum entropy to determine the strength characteristic parameters of the probability density function. Zhang et al. [5] studied the mechanical properties of rocks under the dual action of freezing and melting cycles and surrounding pressure. Mukang et al. [6] studied the relationship between sandstone acoustic emissions and compression deformation by establishing a fine numerical model. L. Gao et al. [7] studied the energy evolution characteristics, based on uniaxial unloading tests, of five different types of rocks, determined their damage rupture thresholds, used energy release and dissipation rates to describe the changes in rock volume and unit strain energy, and concluded that the evolution characteristics of strain energy rates could be easily identified by the crack expansion thresholds. Cheng Hongming et al. [8] found that the energy parameters at the closure stress had a power relationship with the enclosing pressure, while the energy parameters at the initiation stress, damage stress and peak stress had a linear relationship with the enclosing pressure, and the difference between the input energy and elastic strain energy corresponding to the characteristic value points decreased gradually with increase of the enclosing pressure, through graded cyclic loading and unloading tests on sandstones with different enclosing pressures. Ji et al. [9] conducted conventional triaxial compression acoustic emission tests with different circumference pressures to identify rupture precursors for granite. Gong [10] used the acoustic emission frequency analysis algorithm to study precursory signals of rock rupture with instantaneous frequency. Zhang et al. [11] conducted uniaxial compression acoustic emission tests of coal gangue and identified the main frequency and information entropy theory. Wang et al. [12] studied the deformation and strength of three kinds of sandstone under the influence of ambient pressure, using triaxial compression tests. Wang et al. [13] investigated the influence of ambient pressure on shale acoustic emission characteristics through conventional triaxial compression acoustic emission tests. With the continued development of rock engineering, it is particularly important to study rock mechanics and acoustic emission properties under different ambient pressures [13]. The change pattern of acoustic emission signals during rock rupture evolution was studied to reveal its rupture evolution mechanism and explore its rupture precursors [14,15,16,17]. Ye Wanjun et al. [18] studied the changes of fine microstructure and macro-mechanical properties of paleosols under the action of dry and wet cycles. Li Shu-Lin et al. [19] studied the acoustic emission properties before peak intensity of rocks under incremental cycling with unloading. Jiang Jingdong et al. [20] studied the mechanical properties and energy characteristics of mudstone under different water-bearing states. Zhang Yanbo et al. [15,21] studied the acoustic emission spectral characteristics of the rock fracture process by conducting uniaxial compression acoustic emission experiments on water-saturated granite, and extracting the primary and secondary frequencies of acoustic emission signals using fast Fourier transform. Fine sandstone and mudstone are often encountered during coal mining; however, little is known about their associated surrounding pressure effect, acoustic emission characteristics, or rupture precursors. Therefore, in this paper, in order to avoid the fact that the acoustic emission characteristics of single lithology rocks have a chance effect of surrounding pressure and its main rupture precursor information, we adopted a comparative study of different lithology rocks to investigate in depth the mechanical and acoustic emission characteristics of rocks under different surrounding pressure.

Pressure and deformation of the support required for deep rock excavation come from the deformation or rupture of the surrounding rock in the affected area of the excavation roadway. After selecting the appropriate roadway rock support, the surrounding rock is in a three-way force state. With the continuous advancement of the road machine, the surrounding pressure of the roadway rock changes; therefore, the morphology of the surrounding rock and its change status have an important influence on the support characteristics.

## 2. Engineering Background

**Figure 1:**
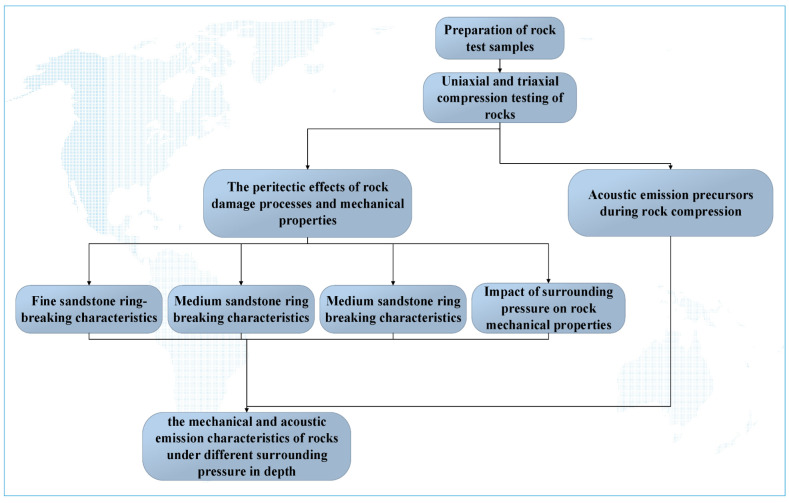
Research Route.

We used a rock sample from the Fucun coal mine, operated by the Zao Mining Group, to analyze mechanical phenomena after the excavation of the roadway and quarry. The downhole of the roadway is located in the southeast of the Dongshi mining area. The roadway is surrounded by the Huancheng fault to the east, the 1009 working face to the west, the Magizhi 1 fault to the south, and the 1008 transport lane under level 3 to the north. Adjacent excavations include the 1009 working face of level 3 to the north of the roadway, the east 8 mining area in the south, and the east 10 mining area in the west. The East Sephuancheng fault is adjacent to the mine. The upper part is the 1009 working face of level 3. The coal seam excavated in this roadway is number 3 of the Shanxi Group; this seam has a stable coal thickness of 2.3–4.5 m (average of 2.5 m) and a simple structure. Three lower coal seams form the original structure of coal; the coal body structure is a strip, while the endogenous fissure is more developed. Closure of the adjacent mined working surface and the excavated roadway did not impact the coal seam structure. The test standard used in the single-axis test part of this paper was DZ/T 0276.18-2015, and the test standard used in the three-axis test part was DZ/T 0276.20-2015. The surrounding rock and lithological characteristics of the 1009 working face of the Fucun coal mine are shown in Table 1 and the research route is shown in Figure 1.

## 3. Test Protocol

### 3.1. Preparation of Rock Test Samples

**Figure 2:**
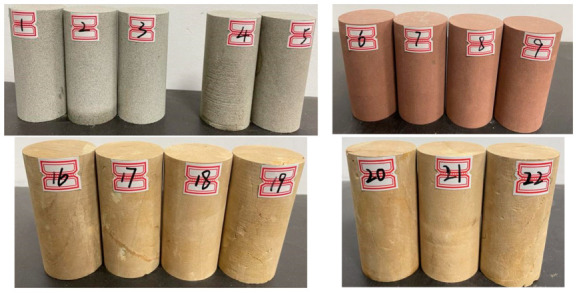
Standard test samples.

The rocks selected for this experiment were sampled in strict accordance with the unified standards to avoid the influence of specimen anisotropy on the experimental results. The rocks were cored by a rock coring machine, and then the specimens were cut and polished to the standard size by a smoothing machine. We used Φ50 mm × 100 mm standard test coal samples [22], which were polished with error controlled at ±0.02 mm [23] (Figure 2).

### 3.2. Test Equipment

To test the rock samples, we used the axial displacement loading method with a simultaneous acoustic emission device to collect the acoustic emission data of the triaxial compression test. The loading equipment was automatically controlled by a full digital computer, which could adopt various load methods (e.g., force, displacement, and axial strain), and could conduct high-speed data collection. It had the advantages of high test accuracy and stable performance.

Uniaxial compression tests were carried out using a microcomputer-controlled o-hydraulic servo universal test machine. The displacement sensor measured the axial relative displacement parameters, and the axial load parameter of the rock sample was measured by a 100-KN load sensor. The data were automatically converted into the corresponding strain and stress for the data acquisition system terminal, and the radial strain of the rock test piece was used to ensure complete data.

**Figure 3:**
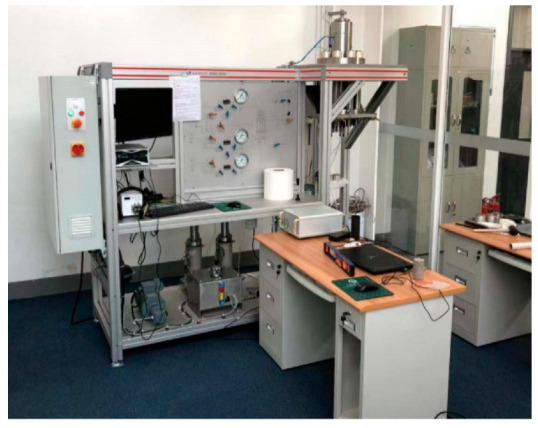
Rock600-50 test system.

**Figure 4:**
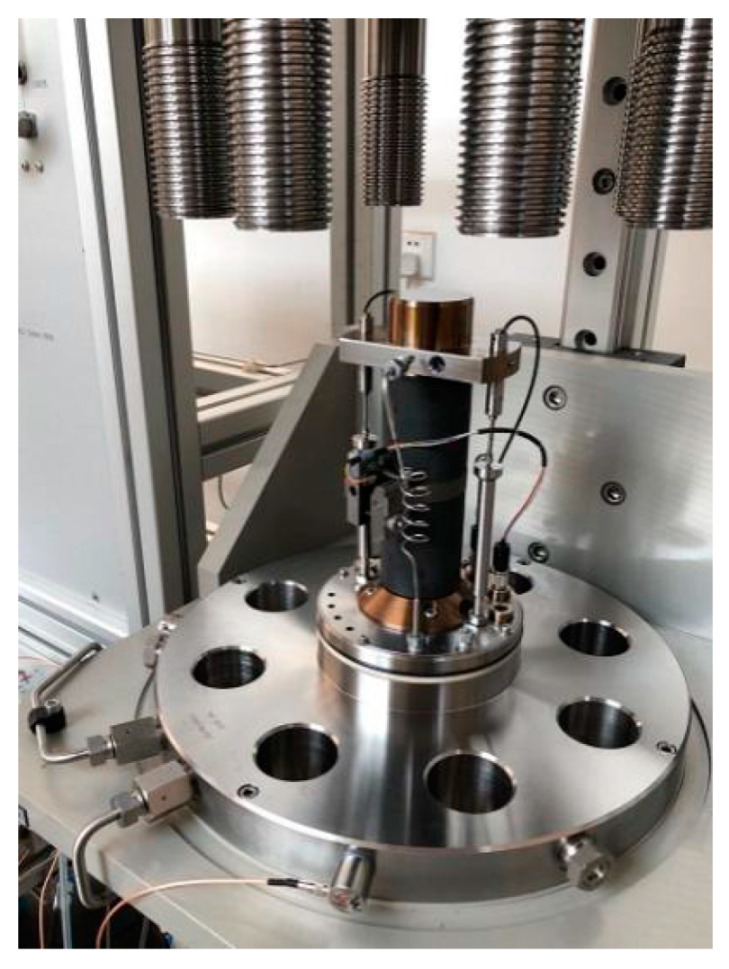
Specimen and sensor apparatus.

Triaxial compression tests were conducted using a Rock600-50 rock three-axial multi-field coupling mechanical test system (Rock600-50 Triaxial and Multi Field Coupling Rock Mechanical Test System; Figure 3) equipped with an acoustic emission positioning test system (Figure 4). The test surrounding pressure was selected according to the lithology (medium sandstone 7 or 11 MPa; fine sandstone, 4, 8, or 12 MPa; mudstone, 1 or 2 MPa).

## 4. Rock Failure and Mechanical Characteristics under Different Surrounding Pressures

### 4.1. Fine Sandstone Ring-Breaking Characteristics

**Figure 5:**
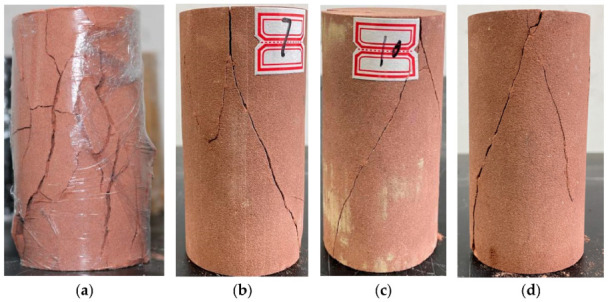
Fine sandstone damage under different confining pressures. Confining pressures of (**a**) 0 MPa, (**b**) 4 MPa, (**c**) 8 MPa, and (**d**) 12 MPa.

The fine sandstone damage characteristics were closely related to the surrounding pressure (Figure 5). When the surrounding pressure was 0 (i.e., a uniaxial compression state), the fine sandstone surface first formed small cracks. With increasing pressure, fracture expansion was followed by transverse crack formation. Finally, owing to loss of carrying capacity, the rock sample failed.

When the surrounding pressure was 4 MPa, the initial crack appeared in the middle of the sample, followed by small cracks on the rock surface. After rock failure, small cracks remained on the rock surface, but there was no obvious transverse crack formation. Compared with 0 MPa, the sample remained relatively complete at the end of the experiment.

At an ambient pressure of 8 MPa, the initial rock crack appeared at the top of the sample; as the axial load increased, microcracks were constantly produced and expanded, and eventually ran through the entire sample resulting in shear damage. Ultimately, the rock showed only small lateral cracks, mainly because the presence of ambient pressure limited the lateral expansion of the fine sandstone.

Under a circumference pressure of 12 MPa, there was an obvious limitation on transverse crack formation. The development angle of rock fissures gradually reduced with increasing surrounding pressure, and there were almost no transverse fissures. The rock mainly suffered from shear damage, and remained mostly complete after completion of the experiment.

In summary, the level of rock damage decreased with increasing surrounding pressure.

### 4.2. Medium Sandstone Ring Breaking Characteristics

**Figure 6:**
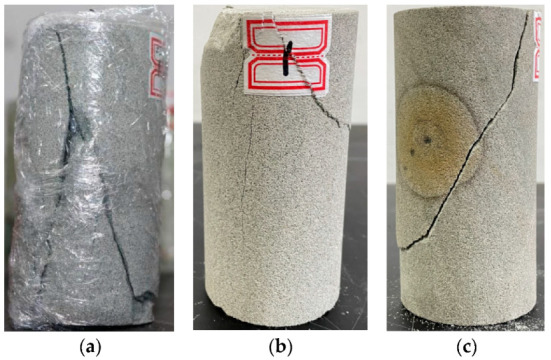
Medium sandstone damage under different confining pressures. Confining pressures of (**a**) 0 MPa, (**b**) 7 MPa, and (**c**) 11 MPa.

The fracture surface of the damage of medium sandstone under different circumferential pressure varied greatly, as shown in Figure 6. Under low circumferential pressure of 0 MPa, the damage of medium sandstone was shown in the formation of many macroscopic fractures, and the damage of uniaxial compression (circumferential pressure = 0 MPa) was serious; the fracture surface was large, and the fracture extension angle was large and close to upright in the case of no lateral constraint or the existence of smaller constraint. In addition to the main fracture, there were also secondary macroscopic fractures and transverse fractures developed, and the main fractures and secondary fractures showed macroscopic cross fractures, which made the rock fragmentation very serious.

With increase of circumferential pressure = 7 MPa, the smaller fractures inside the rock were closed, the development of small fractures reduced, the secondary fractures reduced and were only reflected in subtle places on the rock surface. The crushing of the rock was reduced, compared with the uniaxial state.

The fracture development macroscopically showed a single major fracture and almost no secondary fracture. The dip angle of the fracture surface decreased, the damage angle became smaller, and the rock crushing degree was single.

### 4.3. Mudstone Ring Breaking Characteristics

**Figure 7:**
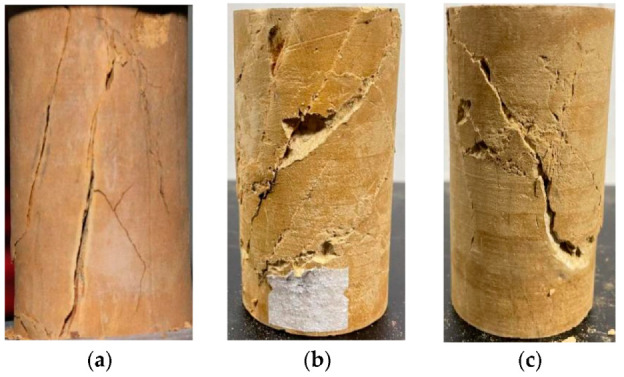
Mudstone damage under different confining pressures. Confining pressures of (**a**) 0 MPa, (**b**) 1 MPa, and (**c**) 2 MPa.

As shown in Figure 7.

In the 0 MPa state, the mudstone had splitting damage, i.e., the damage surface of the mudstone was parallel to the direction of the main compressive stress. The fracture slope was close to vertical, there were more secondary fractures, more minor crushing, more broken blocks, and the degree of damage was serious, mainly because the surrounding pressure was 0, which could not limit the development of transverse fractures in the mudstone.

In the state of 1 MPa, there were still more fractures in the mudstone, but the damage surface was inclined to the direction of the main compressive stress, and the damage of the mudstone changed from splitting damage to shear damage. The degree of fine fragmentation of the mudstone was reduced, compared with that in the state of 0 MPa. However, because of the small circumferential pressure, the development of transverse fractures was greater, and there were still more broken pieces of the rock, although it was reduced compared with the circumferential pressure of 0.

In the state of 2 MPa, the dip angle of the rock rupture fissure increased, under the action of axial stress, a shear slip occurred in the mudstone, and the mudstone was destroyed into monolithic. The enclosing pressure increased, the mudstone was compacted, the degree of fine crushing decreased, the development of a transverse fissure was limited, the degree of fine crushing of rock was greatly reduced, and the larger crushing was almost absent.

### 4.4. Impact of Surrounding Pressure on Rock Mechanical Properties

**Figure 8:**
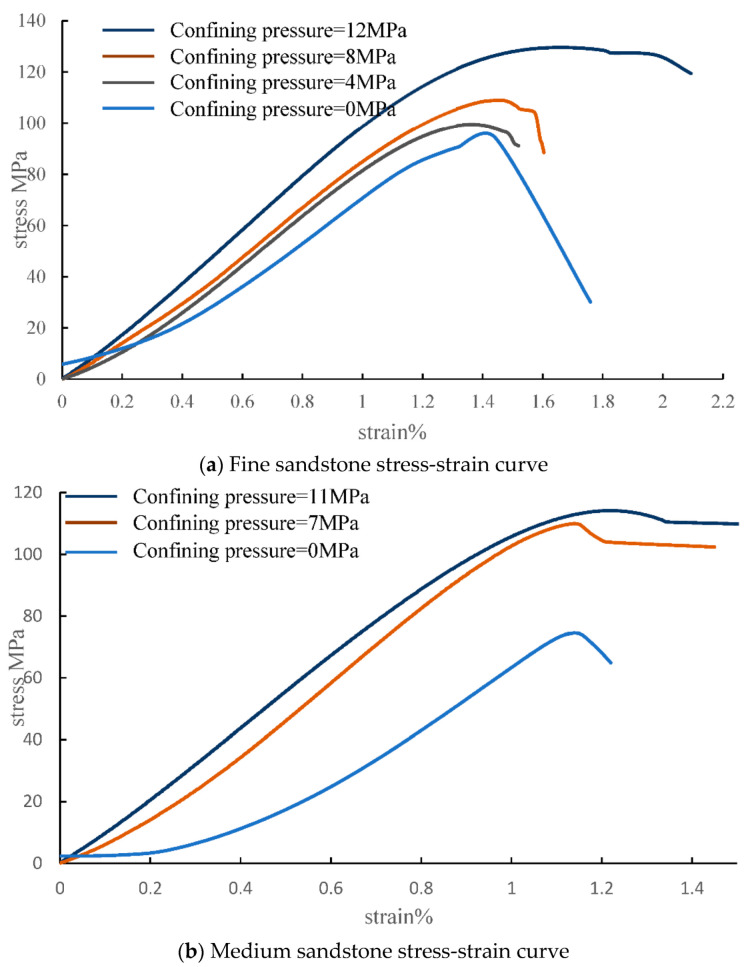

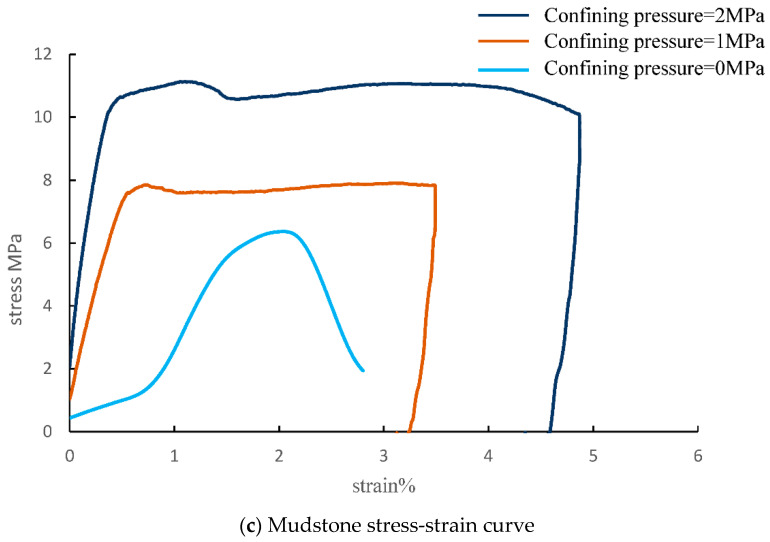
Stress-strain curves of different rock types. (**a**) Fine sandstone, (**b**) medium sandstone, and (**c**) mudstone.

The stress-strain curves of the rock specimens under uniaxial compression are shown in Figure 8. Similar to that of conventional rocks, the uniaxial compression process could be divided into three stages: elasticity, yield, and destruction. In the elastic phase, stress and strain were basically linear, in line with Huke’s law, and had obvious elastic deformation characteristics. In the yield phase, the strain no longer increased linearly with stress, the curve was concave, the rocks began to lose their ability to resist deformation, and underwent irreversible deformation, and gradually changed from elastic to plastic.

According to the test results, it can be seen that the stress-strain results of the three lithologies, fine sandstone, medium sandstone and mudstone, changed in the same trend with increase of the surrounding pressure. The axial stress and axial deformation of the rocks increased and the plastic strain and residual strain also increased significantly. In the uniaxial compression state, the peak compressive strength of the rock was the lowest and the brittle damage was obvious.

At the same time, the peak compressive strength of the rock was closely related to the enclosing pressure and increased with increase of the enclosing pressure, and the compressive strength in the uniaxial state was significantly lower than that in the triaxial state. When the enclosing pressure increased, the strain value of the rock increased by nearly 1.5 times, and the rock needed a longer period of compressive deformation before it was destroyed. The peak compressive strength increased significantly, and the strain before destruction also increased, and the linear elastic phase of the rock accounted for the proportion of the pre-peak axial stress-strain curve segment. The proportion of the rock linear elastic phase in the pre-peak axial stress-strain curve segment gradually increased, the elastic modulus gradually increased, and the increase of the surrounding pressure significantly improved the strain capacity of the rock.

### 4.5. Summary of the Chapter

Comparing the characteristics of uniaxial and triaxial compression damage, it can be seen that all three different lithologies of the rock were severely damaged by uniaxial compression, and the damage of the rock was reduced by increase of the surrounding pressure, while the mechanical properties of the rock were also enhanced with increase of the surrounding pressure. In other words, the damage process and mechanical properties of the rocks are less dependent on the lithology of the rocks.

In addition to the main rupture fracture, there were also many secondary fractures in the uniaxial state of the rock, and the cross damage of the main and secondary rupture fractures made the rock sample break to a serious degree, and the extension and expansion angle of the rock rupture fracture was close to vertical. In the triaxial experiment, the fracture surface of the rock was close to parallel to the main stress direction with a large dip angle in the low circumferential pressure state. The dip angle of the fracture surface of the rock gradually decreased as the circumferential pressure increased, which made the smaller fractures inside the rock compress and close. Comparing the damage patterns of rocks with different surrounding pressure states under the same lithology, with increase of surrounding pressure, the development of transverse fracture extension gradually decreases and the degree of rock damage decreases, which shows that the existence of surrounding pressure restricts the development of transverse fractures in rocks.

Comparing the mechanical characteristics of uniaxial and triaxial compression, we can see that the uniaxial peak compressive strength is the smallest in all three lithologies. The linear elastic stage accounts for the smallest proportion of the pre-peak axial stress–strain curve segment, and the pre-damage strain and peak compressive strength of the rock increases with increase of the enclosing pressure. The reason for this is that in the triaxial compression state, the existence of the enclosing pressure limits the lateral deformation capacity of the rock, and the damage mode of the rock is a single shear damage.

## 5. Acoustic Emission Precursors during Rock Compression

Rock is a natural granular material containing native cracks and pores; under stress, there is crack expansion of internal fractures, pore closure, and plastic deformation, all of which are acoustic emission sources [24]. Generally, the primary pore fracture compression closure phase is very short. In this phase, due to the rock internal microfracture closure, there is resulting small amplitude acoustic emission generation. In the line elastic deformation phase, rock does not produce damaging damage, the ring count is calm. In fracture initiation and the stable expansion phase, the load exceeds the elastic limit of the rock, and the internal fracture begins to rupture and expand, causing the ring count to jump and increase. Each significant increase indicates that there are fissures sprouting or rupture in the specimen. In the non-stable extension stage of fracture, the ringing count increases sharply and intensively, which is due to the rapid fracture expansion when the main rupture occurs, and the original fracture penetration, thus developing into a fracture network, leading to the formation of macro rupture [25].

**Figure 9:**
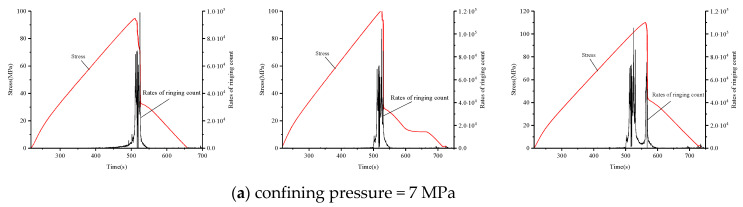

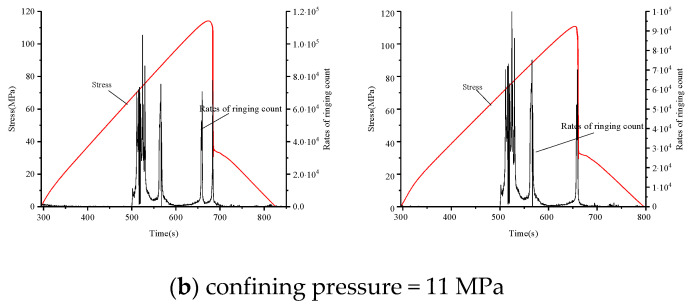
Count rate, stress, and time relationship of medium sandstone failure under different confining pressures. Confining pressure of (**a**) 7 MPa and (**b**) 11 MPa.

**Figure 10:**
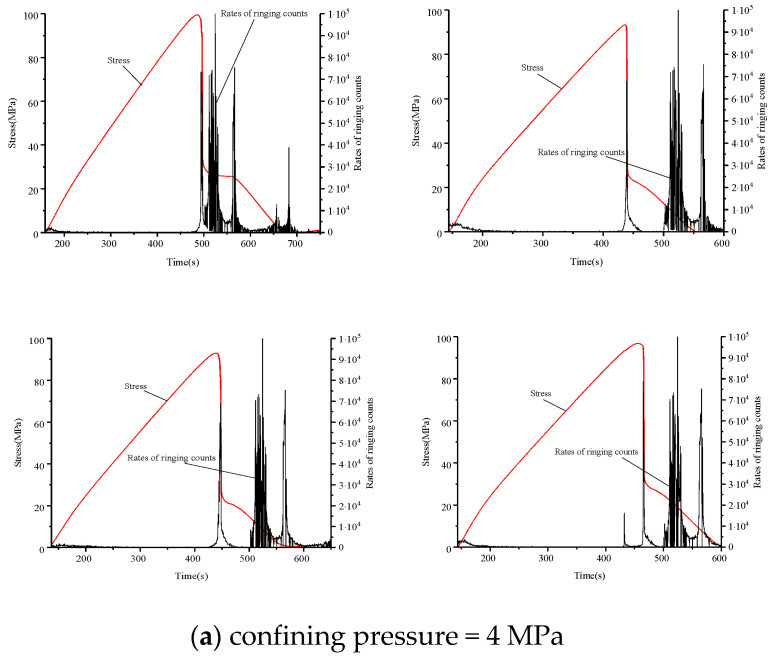

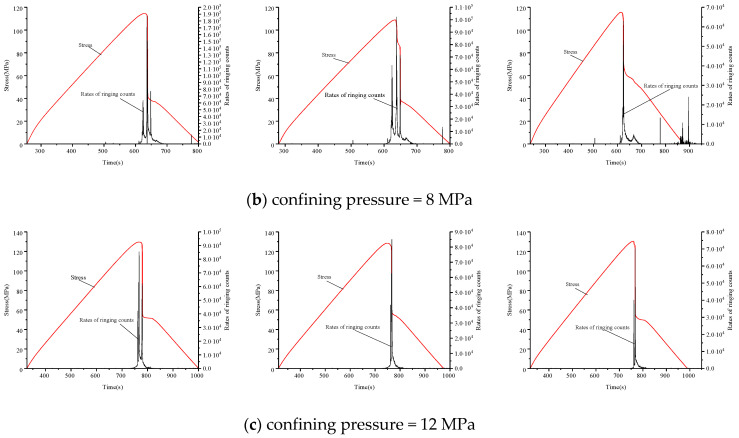
Count rate, stress, and time relationship of fine sandstone failure under different confining pressures. Confining pressure of (**a**) 4 MPa, (**b**) 8 MPa, and (**c**) 12 MPa.

**Figure 11:**
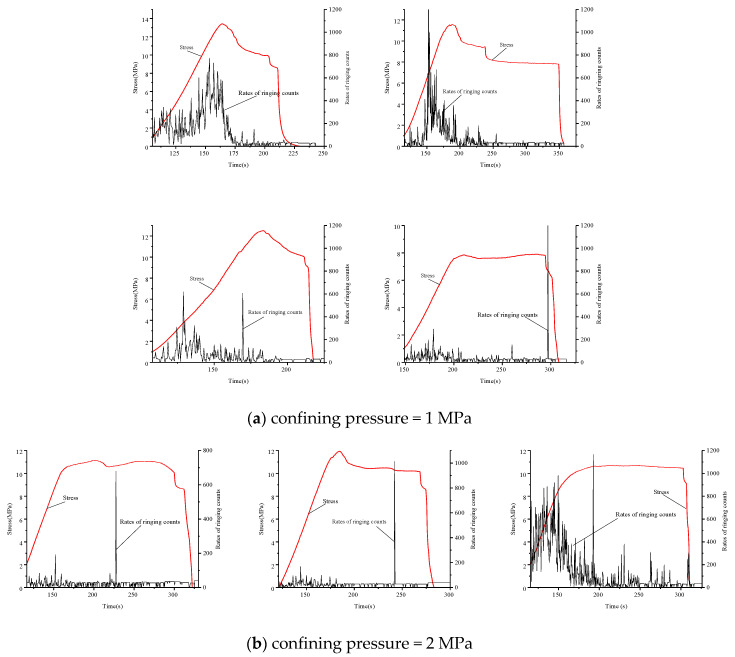
Count rate, stress, and time relationship of mudstone failure under different confining pressures. Confining pressure of (**a**) 1 MPa and (**b**) 2 MPa.

Acoustic emission tests were conducted using a displacement load; that is, the data changed with loading time. An acoustic emission device was used to analyze the relationship between acoustic emission and rock deformation characteristics. The main stress-time-bell count ratio curves for different ambient pressure levels for medium sandstone, fine sandstone, and mudstone are shown in Figure 9, Figure 10, and Figure 11, respectively.

As shown in Figure 9, the development of the ringing count rate-time in the main stress-time-bell count rate curve can be divided into three stages: destruction, surge, and calm. When the surround pressure was 7 MPa, the end point of the calm phase was near the peak stress of 90%. When the circumference pressure increased to 11 MPa, the end point occurred before the peak stress, consistent with the elastic stage of the stress-strain curve. During the internal rock crack initiation stage, acoustic emission activity was low, with only a few cracks and small bell count rate.

During the surge phase there was a rapid expansion of internal cracks, consistent with the yield phase of the stress-strain curve; as the stress increased, internal cracks constantly expanded and propagated, and the acoustic emission events increased accordingly (i.e., bell counting rate increased to the maximum value). At a pressure of ~7 MPa, the surge phase occurred after the peak rock stress. As the surrounding pressure increased to 11 MPa, the surge phase occurred at 70–80% of peak stress (i.e., prior to the peak). Therefore, the beginning of the bell counting rate also indicated the peak stress (i.e., the point at which damage occurred). In other words, a sudden increase in the bell count rate could be used as a precursor of rock damage.

The rock destruction stages for the different surrounding pressures corresponded to the destruction stages of the stress-time curve. Medium sandstone destruction occurred immediately after reaching the peak stress; compared with the other rock types, it had fewer acoustic emission events and a smaller bell counting rate.

For the fine sandstone, we observed a decreasing rock bell count rate during all three stages with increasing circumference pressure (Figure 10). The calm stages for surrounding pressures of 4, 8, and 12 MPa all occurred before the peak stress, because the surrounding pressure removed the small crack pressure inside the rock.

In the surge phase, the rock began to undergo irreversible deformation, consistent with the yield phase of the stress-strain curve, and cracks began to form; rock damage occurred after reaching peak stress. At the same time, acoustic-emission events became active; the bell count rate increased substantially, compared with the calm phase. For a surrounding pressure of 4 MPa, the surge phase began in the destruction phase, while for a surrounding pressure of 8 MPa it began near the peak stress. In contrast, for a surrounding pressure of 12 MPa, the surge phase occurred close to the peak stress. The sudden increase in the count rate of fine sandstone acoustic emission ringing under high ambient pressure could be used as a precursor to rock damage.

The damage stage occurred over a short period after the peak stress decreased rapidly. Owing to the surrounding pressure, samples did not fail immediately. First, a large number of internal cracks began to expand until they ran all through the rock; while, at the same time, the acoustic and emission events were large.

For the mudstone, the calm phase was distributed near the peak stress (Figure 11); the rock produced a small bell count rate before the peak. The spike phases for all surrounding pressures occurred at 70–100% peak stress, consistent with the yield phase of the stress-strain curve. The damage stage occurred after the peak stress, after which cracks developed until the mudstone failed completely and the bell count rate fell.

Increased surrounding pressure significantly reduced the bell count rate of the mudstone; moreover, as the timings of the three stages were similar for surrounding pressures of 1 and 2 MPa, the bell count rates were also similar. Increasing bell count rate indicated the impending failure of the mudstone.

## 6. Conclusions

In this study, we performed triaxial and uniaxial compression experiments to analyze the deformation and damage characteristics of fine sandstone, medium sandstone and mudstone under different surrounding pressure. The results show that:

The surrounding pressure effects of rock damage processes and mechanical properties are less correlated with rock lithology. The presence of confining pressure leads to the closure of smaller internal cracks, limiting the lateral deformation capacity of the rock, which has a significant impact on the deformation and damage characteristics of the rock. The damage mode of rocks with higher confining pressure is single shear damage. Increasing the confining pressure reduces the development of transverse cracks and increases the compressive strength of the rock.

The acoustic emission ringing count rate reflects the development of fractures within the rock. As the surrounding pressure increases, the acoustic emission ringing count rate decreases as small cracks close. When the acoustic emission ring count rate is small, crack production decreases. The increase of the surrounding pressure makes the fine fractures in the rock destruction process tight and closed, so the acoustic emission ringing count rate of the rock also decreases with the increase of the rock surrounding pressure, and it also further proves the limitation effect of the surrounding pressure on rock fracture development.

A relationship exists between the acoustic emission pattern and the mechanical properties of the rock. A high-frequency band of acoustic emission ringing rate appears in the yielding phase of the stress-strain curve, and in triaxial tests, the ringing rate of the rock increases abruptly before and after the stress peak. In summary, surge in the ringing count rate is significant and can be used as a precursor to rock damage.

## Figures and Tables

**Table 1:** Comprehensive histogram of working face conditions.

Thickness (m)	Depth (m)	Formation
1.85	52.66	Medium sandstone
9.0	61.66	Fine sandstone
10	71.66	Medium sandstone
5	76.66	Siltstone
0.3	76.96	Mudstone
5.5	82.46	coal seam
0.3	82.76	Mudstone
5.12	87.88	Siltstone
0.15	88.03	Mudstone

## Data Availability

The data used to support the findings of this study are available from the corresponding author upon request.
